# Cephalometric characteristics of individuals with impacted maxillary canines regarding their impaction sector: A comparative study

**DOI:** 10.4317/jced.63422

**Published:** 2025-11-30

**Authors:** Hugo Rodrigo Mendoza-Trujillo, Yalil Augusto Rodríguez-Cárdenas, Gustavo Armando Ruíz-Mora, Pedro Luis Tinedo-López, Luis Ernesto Arriola-Guillén

**Affiliations:** 1 Posgraduate student, Universidad Científica del Sur, Lima, Perú. ORCID ID: 0000-0003-0432-21142; 2 Ph.D. and Associate Professor of the Division of Oral and Maxillofacial Radiology, School of Dentistry, Universidad Nacional de Colombia, Bogotá, Colombia. ORCID ID: 0000-0002-3107-3013; 3 Ph.D. and Associate Professor of the Division of Orthodontics, Faculty of Dentistry, Universidad Nacional de Colombia, Bogotá D.C, Colombia. ORCID ID: 0000-0002-9954-1047; 4 MSc. and Associate Professor of the Division of Implantology, Universidad Científica del Sur, Lima, Perú. ORCID ID: 0000-0002-2102-4437; 5 Ph.D. and Associate Professor of the Division of Orthodontics, Universidad Científica del Sur, Lima, Perú. ORCID ID: 0000-0003-0010-5948

## Abstract

**Background:**

Impacted maxillary canines (ICM) can significantly change the shape of the anterior maxilla, particularly when they are situated near the dental midline. This research aimed to evaluate the cephalometric characteristics of individuals with ICM considering their impaction sector.

**Material and Methods:**

This retrospective cross-sectional study included 135 sets of panoramic and lateral head radiographs divided into three groups. Group A consisted of 45 patients with IMC in sectors 1, 2, and 3. Group B included 45 patients with IMC in sectors 4 and 5, and the control group, matched for sex and age, comprised 45 patients without IMC. Cephalometric measurements were collected using WebCeph software, focusing on variables such as the impaction sector, growth pattern, SNA angle, Wits appraisal, 1/NA angle, 1-NA distance, nasolabial angle, and maxillary length. One-way ANOVA with Tukey's post hoc test and multiple linear regression analyses were used for group comparisons. (P&lt; 0.05).

**Results:**

Statistically significant differences were observed in the SNA angle, with the impaction group in sectors 4 and 5 showing greater values (82.96°) compared to the control group (81.19°; p&lt;0.05). Additionally, the 1-NA distance was smaller in sectors 1, 2, and 3 (2.96 mm) than in the control group (4.20 mm; p&lt;0.05). Furthermore, the nasolabial angle was greater in sectors 4 and 5 (102.22°) than in the control group (93.70°; p&lt;0.05).

**Conclusions:**

Patients with IMCs show a distinct cephalometric pattern, including an increased SNA angle, especially when the impaction is near the midline. This condition is associated with incisor retrusion and a higher nasolabial angle. These findings suggest that the location of canine impaction affects both the dentoskeletal structure and the soft tissue profile.

## Introduction

Impacted maxillary canine (IMC) is a dental developmental anomaly with a prevalence of 0.8% and 3% in the general population. This condition can lead to significant functional and aesthetic issues, including displacement of adjacent teeth, root resorption of incisors, and disturbances in maxillofacial development. Therefore, early diagnosis is crucial for facilitating traction, reducing treatment time, and minimizing adverse effects (1 - 5). Thus, the sagittal position of the impacted canine is a vital factor in treatment planning, and it can be assessed using the impaction sector classification proposed by Ericson and Kurol (6 - 9). Maxillary canine impaction may alter the morphology of the anterior maxilla, especially when the impacted canines are positioned near the dental midline (8 , 10). This condition can also affect the positioning of the central incisors in the premaxilla. However, these differences should be demonstrated through cephalometric analysis comparing groups with various impaction sectors or a control group (10 - 12). Although prior studies have explored skeletal characteristics in patients with impacted canines, (12 - 14) none have specifically compared these characteristics based on their impaction sector. Additionally, most existing research has focused on populations in Europe and North America, highlighting the need for more studies on diverse populations to compare findings (13 - 20). In this way, this study aimed to compare the cephalometric characteristics of individuals with IMC considering their impaction sector. The research aims to improve our understanding of the dentoalveolar, and skeletal factors involved in canine impaction and contribute to developing early diagnostic strategies that enhance orthodontic and surgical planning.

## Material and Methods

**Figure 1:**
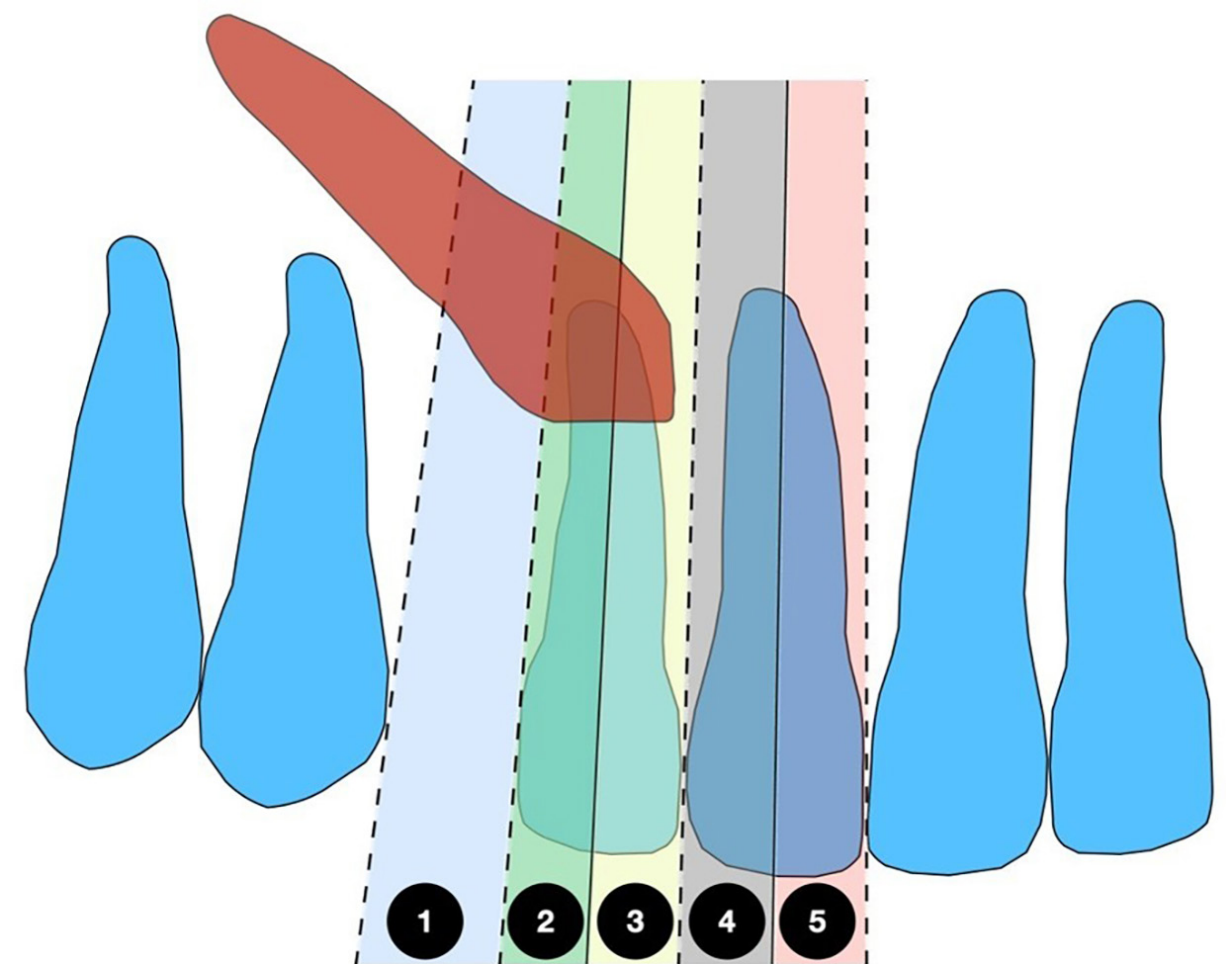
Maxillary canine impaction sector by Ericson and Kurol classification.1 from the mesial aspect of the first premolar to the distal aspect of the lateral incisor. (sector 1). From the distal aspect of the lateral incisor to the long axis of the lateral incisor (sector 2). From the long axis of the lateral incisor to the distal aspect of the central incisor (sector 3). From the distal aspect of the central incisor to the long axis of the central incisor (sector 4). From the long axis of the central incisor to the interincisor median line (sector 5).

- Study characteristics and ethical approval This observational, cross-sectional, retrospective study received approval from the Científica del Sur University Ethics Committee in Lima, Peru (protocol number: POS-53-2025-00308). The study adhered to the STROBE (Strengthening the Reporting of Observational Studies in Epidemiology) guidelines and followed the principles of the Declaration of Helsinki. No interventions were performed on patients, nor was any new clinical or personal data collected. All radiographic images were anonymized to ensure confidentiality and compliance with ethical standards. - Sample size and population The study sample included 135 panoramic and lateral head radiographs from young adult patients who visited a radiology center in Lima, Peru. The participants were divided into three groups. Group A comprised 45 patients with IMC in sectors 1, 2, and 3. Group B included 45 patients with IMC in sectors 4 and 5. The control group, matched for sex and age, consisted of 45 patients without IMC. The sample size was calculated using Stata/SE 18.0 (StataCorp LLC, College Station, TX, USA) with a power of 90% and a significance level of 5%, based on the comparative means reported in the study by Cernochova et al. (4). The minimum required sample size for each group was 40 participants. - Inclusion criteria The inclusion criteria consisted of high-quality digital lateral cephalometric and panoramic radiographs of young adult patients aged 13 to 40 years who had IMC. We excluded radiographs from patients who had undergone orthodontic treatment, presented with supernumerary teeth, odontomas, cysts, or had any syndromes, cleft lip/palate, a history of maxillary trauma, or missing maxillary incisors. - Calibration and observer agreement Two trained and calibrated observers conducted all measurements. The training was supervised by an orthodontist with over 10 years of experience. Additionally, inter-observer agreement for qualitative variables was evaluated using Cohen's Kappa test, while intra-observer reliability was assessed using the Intraclass Correlation Coefficient (ICC). - Cephalometric Measurements Cephalometric analyses were conducted using WebCeph software (AsyRaM Inc., 2025). Each patient was registered along with their lateral cephalometric and panoramic radiographs. The investigator reviewed the software's AI-assisted cephalometric tracing for accuracy. The radiographic scale marker located in the upper right corner of the lateral cephalogram was used for calibration (Table 1, Fig. 1).

Table 1

1

- Statistical analyses The biostatistical analysis was conducted using Stata/SE 18.0. The mean and standard deviation (SD) were calculated for each variable. The Shapiro-Wilk test was employed to assess normality for the bivariate analysis. If normality was confirmed, ANOVA and Tukey post hoc tests were performed to compare outcome variables comparing three groups, with a significance level at p &lt; 0.05.

## Results

- Reliability The Cohen's Kappa test values were 1 for intra and inter-observer reliability. The ICC values ranged from 0.90-1.00 in the intra-observer reliability for all evaluations. The error method was less than 1 mm. - Main results Table 2 shows the initial characteristics of the sample, highlighting the similarity among the three groups in terms of the Wits appraisal (p=0.876) and skeletal relationship (p=0.195).

Table 2

However, the control group was older (p&lt;0.001), and significant differences were observed in the distribution of impaction sectors across the groups (p&lt;0.001). Table 3 presents the cephalometric characteristics of the anterior maxilla, comparing the three groups with and without canine impaction.

Table 3

Significant differences were found in the SNA angle between the control group (81.19°) and the group with impaction in sectors 4 and 5 (82.96°). The position of the upper central incisor (1-NA) in the control group was 4.20mm, while in the impaction groups it was lower, showing statistical significance only in sectors 1, 2, and 3 (2.96mm). The nasolabial angle was smaller in the control group (93.70°) compared to both impaction groups, with the highest value observed in sectors 4 and 5 (102.22°). Table 4 presents three multiple linear regression models evaluating the influence of predictive variables (age, sex, impaction group, and skeletal relationship) on incisor position and inclination, as well as on the nasolabial angle, but no significant associations were found (p&gt;0.05).

Table 4

## Discussion

Maxillary canine impaction is an anomaly that affects dental occlusion, maxillary development, and dental esthetics; for this reason, it is studied in orthodontics to improve early diagnosis and long-term prognosis. Beyond this, the clinical and radiographic characteristics of individuals with impacted maxillary canines (IMC), compared to a control group, have been scarcely reported in the literature and even less so when considering the sector of impaction, which may alter certain cephalometric landmarks, particularly in the anterior region. In this context, the present study compared the cephalometric characteristics from lateral head radiographs of patients with IMC in different sectors of impaction, with the aim of identifying whether differences exist in the dentoskeletal patterns of patients presenting this anomaly across different sectors of impaction, along with a control group. The results of this study showed a statistically significant difference in SNA angle measurements (p&lt; 0.05). An increase in this angle was found in the IMC groups compared to the control group. Radiographs with IMC in sectors 4 or 5 showed significantly greater SNA values than those with impaction in sectors 1, 2, or 3. This finding indicates that individuals with IMC exhibit a protruded premaxilla, which is more pronounced in cases of impaction near the midline. Similar results were reported by Ciavarella et al. (20), who found an increase in the SNA angle in patients with IMC when comparing craniofacial morphology to a control group. Although that study did not consider the sector of impaction, its findings align with the present analysis. Likewise, Cernochová et al. (4) also reported greater SNA values in patients with IMC. Furthermore, Athanasiou et al. (18) using three-dimensional morphometric analysis demonstrated greater sagittal projection of the premaxilla in IMC cases, which is consistent with an increased SNA angle within a conventional cephalometric framework. The findings of the present study confirm an increased SNA angle in patients with IMC and suggest greater severity in those with impaction in sectors 4 and 5, probably because most of these midline-closer impactions are buccal and, therefore, exert direct influence on point A. The results from the comparison of the 1-NA distance also showed significant differences. Contrary to the increase observed in the SNA angle with the presence of IMC, the 1-NA distance was reduced in the group with impaction in sectors 1, 2, and 3, indicating greater retrusion of the upper incisors. Cernochová et al. (4) reported a reduced 1-NA distance in the IMC group with palatal projection. More retroclined incisors were found compared to the control group. These findings help define a cephalometric pattern regarding the position of the upper incisors in patients with this anomaly. Regarding the nasolabial angle, a significant increase was observed in the IMC groups. This finding may be directly related to the position of the upper incisor, as dentoalveolar retrusion can displace the upper lip posteriorly and, consequently, increase the nasolabial angle. Although this association between the nasolabial angle and IMC has not been described in the literature, an orthodontic relationship between lip projection and the position and inclination of the incisors does exist. This finding represents a new contribution to literature by proposing the nasolabial angle as a useful cephalometric parameter in the assessment of IMC cases. The findings of this study enhance our understanding of the cephalometric patterns in patients with internal maxillary constriction (IMC). We observed an increased SNA angle, with greater values noted in sectors 4 and 5. Additionally, there was a reduction in the 1-NA distance, suggesting greater upper incisor retrusion in impactions located in sectors 1, 2, and 3. These results may indicate a dentoalveolar compensation related to the site of impaction. Additionally, we observed an increase in the nasolabial angle, a finding not previously documented in the literature regarding IMCs. This increase is a potential factor for aesthetic and cephalometric evaluation in patients with this condition. Limitations This study did not include the complete clinical records of the patients whose radiographs were analyzed. This limitation prevented a broader generalization of the results. It is recommended that future studies collect full clinical records to allow for a deeper analysis of the findings and complement this research with longitudinal studies to observe whether these features resolve following canine traction.

## Conclusions

Patients with IMCs exhibit a modified cephalometric pattern, characterized by an increased SNA angle more pronounced when the impaction is located near the midline and incisor retrusion, as evidenced by the reduced 1-NA distance. Additionally, an increased nasolabial angle was observed, likely related to the incisor retrusion described above. These findings suggest that the sector of canine impaction influences both the dentoskeletal pattern and soft tissue profile, which is relevant for the diagnosis and treatment planning of this dental anomaly.

## Figures and Tables

**Table 1:** Definitions of the measurements used in this research.

Variable	Definition
IMC sector	The location sector of the impacted maxillary canine by Erikson and Kurol classification1
Growth pattern parameters	
GoGnSN	The angle between the mandibular plane (Gonion (Go) Gnation (Gn)) and Sella-Nasion line (SN).
FOPSN	The angle between the functional occlusal plane (FOP) and Sella-Nasion line (SN).
Y axis SN	The angle between the Sella-Nasion line (SN) and the Sella-Gnation line (SGn).
Skeletal, dental and soft tissue parameters	
SNA	The angle between Sella (S), Nasion (N) and subnasal (A).
Wits appraisal	Anteroposterior skeletal relationship between the maxilla and mandible.
1/NA	The angle between the long axis of the maxillary central incisor and the Nasion (N) subnasal (S) line.
1-NA	The distance from the most anterior point of the maxillary central incisor to the Nasion (N) subnasal (A) line.
Nasolabial angle	The angle between the upper lib line and the columella line.
Effective length of maxilla	The distance from Condylion (Co) to subnasal (A).

1

**Table 2:** Baseline characteristics of the sample.

Group	n	Age				p		
		Mean	SD	Min.	Max.			
Control	47	28.52 a	5.45	20	40	<0.001+		
Canine impaction (sectors 1, 2 and 3)	47	19.03 b	5.48	9	30			
Canine impaction (sectors 4 and 5)	40	20.71 b	8.36	11	48			
Group	n	Wits appraisal				p		
		Mean	SD	Min.	Max.			
Control	47	1.77 mm a	0.81	1	3	0.876 +		
Canine impaction (sectors 1, 2 and 3)	47	1.83 mm a	0.92	1	3			
Canine impaction (sectors 4 and 5)	40	1.93 mm a	0.89	1	3			
Group		Skeletal classification				p		
		1	2	3	Total			
Control	n	29	6	12	47	p= 0.195 ++		
	%	61.7	12.8	25.5	100			
Canine impaction (sectors 1, 2 and 3)	n	28	9	10	47			
	%	59.6	19.1	21.3	100			
Canine impaction (sectors 4 and 5)	n	24	12	4	40			
	%	60	30	10	100			
Total	n	81	27	26	134			
	%	60.4	20.1	19.4	100			
Group		Canine impaction sector						p
		1	2	3	4	5	Total	
1	n	9	19	19	0	0	47	
	%	19.1	40.4	40.4	0	0	100	<0.001 ++
2	n	0	0	0	21	19	40	
	%	0	0	0	52.5	47.5	100	

+ ANOVA test, Tukey post hoc; different letters indicate statistically significant differences.++ Chi-square test.

**Table 3:** Cephalometric characteristics in the evaluated groups.

Group	Measurement	n	Mean	SD	Min.	Max.
Control	SNA angle	47	81.19° a	2.05	75.95	84.34
	Incisor inclination (1/NA)	47	21.40° a	8.22	-2.10	39.02
	Incisor position (1-NA)	47	4.20 mm a	2.45	0.05	10.73
	Nasolabial angle	47	93.70° ab	12.32	69.86	119.04
	Maxillary length (CoA)	47	79.85mm a	5.70	57.49	93.15
Canine impaction in sectors 1, 2 and 3	SNA angle	47	81.96° ab	4.11	75.62	99.11
	Incisor inclination (1/NA)	47	21.18° a	6.44	7.15	35.10
	Incisor position (1-NA)	47	2.96 mm bc	2.09	0.07	7.10
	Nasolabial angle	47	102.22° c	11.72	73.04	121.47
	Maxillary length (CoA)	47	78.18 mm a	4.98	66.54	90.61
Canine impaction in sectors 4 and 5	SNA angle	40	82.96° bc	3.39	75.63	90.54
	Incisor inclination (1/NA)	40	20.46° a	7.20	8.70	35.22
	Incisor position (1-NA)	40	3.15 mm ab	2.08	0.31	9.93
	Nasolabial angle	40	95.27° a	15.03	67.31	124.87
	Maxillary length (CoA)	40	78.63 mm a	5.36	69.14	89.03

ANOVA and Tukey post hoc test.Different letters indicate statistically significant differences (p<0.05).

**Table 4:** Linear regression evaluating the influence of predictive variables on upper central incisor position and inclination, and the nasolabial angle.

Predictor variable	p	95.0% Confidence interval for B	
		Lower limit	Upper limit
	Incisor position (1-NA)		
Constant	0.076	-0.346	6.782
Age	0.295	-0.056	0.181
Sex	0.223	-2.313	0.552
Canine impaction group	0.901	-1.008	0.891
Skeletal classification	0.471	-1.149	0.539
	Incisor inclination (1/NA)		
Constant	0.001	9.052	33.753
Age	0.643	-0.316	0.507
Sex	0.417	-6.985	2.942
Canine impaction group	0.462	-2.075	4.506
Skeletal classification	0.491	-3.935	1.913
	Nasolabial angle		
Constant	0.000	67.914	106.940
Age	0.527	-0.444	0.857
Sex	0.168	-2.383	13.302
Canine impaction group	0.474	-7.068	3.330
Skeletal classification	0.740	-5.388	3.853

4

## Data Availability

The datasets used and/or analyzed during the current study are available from the corresponding author.

## References

[1] Ericson S, Kurol J (1988). Early treatment of palatally erupting maxillary canines by extraction of the primary canines. Eur J Orthod.

[2] Proffit WR (1991). Skinner’s 2 Science of Dental Materials.

[3] Ericson S, Kurol PJ (2000). Resorption of incisors after ectopic eruption of maxillary canines: a CT study. Angle Orthod.

[4] Cernochova P, Izakovicova-Holla L (2012). Dentoskeletal characteristics in patients with palatally and buccally displaced maxillary permanent canines. Eur J Orthod.

[5] Arriola-Guillén LE, Ruíz-Mora GA, Rodríguez-Cárdenas YA, Aliaga-Del Castillo A, Boessio-Vizzotto M, Dias-Da Silveira HL (2019). Influence of impacted maxillary canine orthodontic traction complexity on root resorption of incisors: A retrospective longitudinal study. Am J Orthod Dentofacial Orthop.

[6] Arriola-Guillén LE, Ruíz-Mora GA, Rodríguez-Cárdenas YA, Aliaga-Del Castillo A, Dias-Da Silveira HL (2018). Root resorption of maxillary incisors after traction of unilateral vs bilateral impacted canines with reinforced anchorage. Am J Orthod Dentofacial Orthop.

[7] Cicek O, Gurel T, Demir Cicek B (2023). Investigation of the Relationship of Impacted Maxillary Canines with Orthodontic Malocclusion: A Retrospective Study. Children (Basel).

[8] Arriola-Guillén LE, Aliaga-Del Castillo A, Ruíz-Mora GA, Rodríguez-Cárdenas YA, Dias-Da Silveira HL (2019). Influence of maxillary canine impaction characteristics and factors associated with orthodontic treatment on the duration of active orthodontic traction. Am J Orthod Dentofacial Orthop.

[9] Guarnieri R, Cavallini C, Vernucci R, Vichi M, Leonardi R, Barbato E (2016). Impacted maxillary canines and root resorption of adjacent teeth: A retrospective observational study. Med Oral Patol Oral Cir Bucal.

[10] Chávez-Alvarez C, Arriola-Guillén LE, Rodríguez-Cárdenas YA, Ruíz-Mora GA, Fiori-Chincaro G, Dias-Da Silveira HL (2019). Changes in maxillary incisor inclination and position after traction of unilateral vs bilateral maxillary impacted canines in nonextraction treatment: A cone-beam computed tomography study. Am J Orthod Dentofacial Orthop.

[11] Arboleda-Ariza N, Schilling J, Arriola-Guillén LE, Ruíz-Mora GA, Rodríguez-Cárdenas YA, Aliaga-Del Castillo A (2018). Maxillary transverse dimensions in subjects with and without impacted canines: A comparative cone-beam computed tomography study. Am J Orthod Dentofacial Orthop.

[12] Baidas LF, Alshihah N, Alabdulaly R, Mutaieb S (2022). Severity and Treatment Difficulty of Impacted Maxillary Canine among Orthodontic Patients in Riyadh, Saudi Arabia. Int J Environ Res Public Health.

[13] Jung YH, Liang H, Benson BW, Flint DJ, Cho BH (2012). The assessment of impacted maxillary canine position with panoramic radiography and cone beam CT. Dentomaxillofac Radiol.

[14] Grandhi RK, Tira DE (2003). Prediction of maxillary canine impaction using sectors and angular measurement. Am J Orthod Dentofacial Orthop.

[15] Alfaleh W, Al Thobiani S (2021). Evaluation of impacted maxillary canine position using panoramic radiography and cone beam computed tomography. Saudi Dent J.

[16] Amini F, Hamedi S, Haji Ghadimi M, Rakhshan V (2017). Associations between occlusion, jaw relationships, craniofacial dimensions and the occurrence of palatally-displaced canines. Int Orthod.

[17] Mercuri E, Cassetta M, Cavallini C, Vicari D, Leonardi R, Barbato E (2013). Skeletal features in patient affected by maxillary canine impaction. Med Oral Patol Oral Cir Bucal.

[18] Athanasiou M, Papadopoulou CI, Alamoudi R, Halazonetis D, Verna C, Gkantidis N, Kanavakis G (2024). Palatal canine impaction is associated with craniofacial shape in humans. Eur J Orthod.

[19] Al Balbeesi HO, Al Kawari HM, Al Tamimi AS, Al Mubarak I, Al Ibrahim KI, Divakar DD (2020). Association Between Canine Impaction and Skeletal Pattern in the Sagittal and Vertical Planes. Int J Periodontics Restorative Dent.

[20] Ciavarella D, Lorusso M, Leone M, Ferrara D, Fanelli C, Illuzzi G, Ortu E, Lo Muzio L, Tepedino M (2024). Craniofacial morphology in patients with impacted canine: a case control-study. Minerva Dent Oral Sci.

